# Advancing Fluoride-Ion Batteries with a Pb-PbF_2_ Counter Electrode and a Diluted Liquid Electrolyte

**DOI:** 10.1021/acsenergylett.3c02228

**Published:** 2023-12-08

**Authors:** Giulia Galatolo, Omar Alshangiti, Camilla Di Mino, Guillaume Matthews, Albert W. Xiao, Gregory J. Rees, Maximilian Schart, Yvonne A. Chart, Lorenz F. Olbrich, Mauro Pasta

**Affiliations:** † Department of Materials, University of Oxford, Oxford, OX1 3PH, United Kingdom; ‡ The Faraday Institution, Harwell Campus, Quad One, Becquerel Avenue, Didcot OX11 0RA, United Kingdom

## Abstract

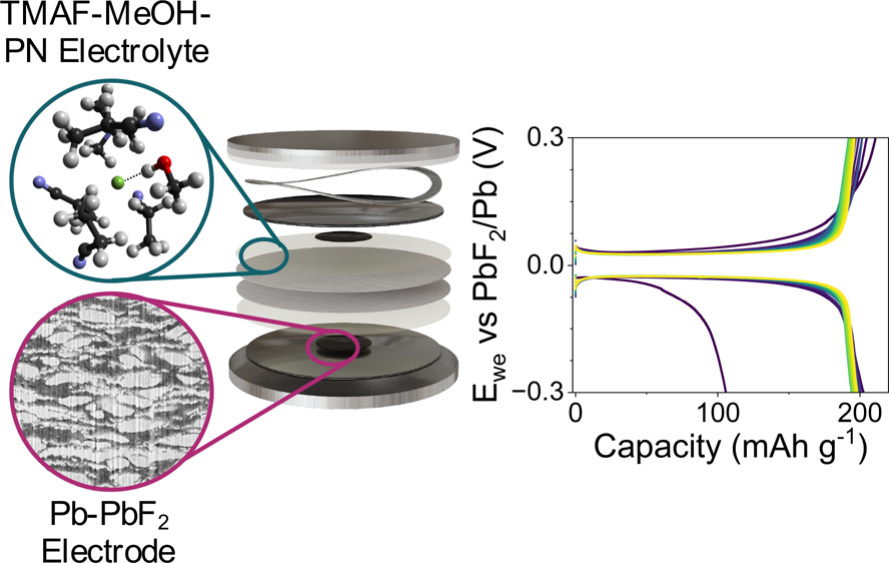

Fluoride ion batteries (FIB) are a promising post lithium-ion technology thanks to their high theoretical energy densities and Earth-abundant materials. However, the flooded cells commonly used to test liquid electrolyte FIBs severely affect the overall performance and impede comparability across different studies, hindering FIB progress. Here, we report a reliable Pb-PbF_2_ counter electrode that enables the use of two-electrode coin cells. To test this setup, we first introduce a liquid electrolyte that combines the advantages of a highly concentrated electrolyte (tetramethylammonium fluoride in methanol) while addressing its transport and high-cost shortcomings by introducing a diluent (propionitrile). We then demonstrate the viability of this system by reporting a BiF_3_–Pb-PbF_2_ cell with the highest capacity retention to date.

Achieving net-zero emissions by 2050 relies on the electrification of various sectors. This in turn requires batteries with higher energy densities which are free from expensive and critical battery minerals such as cobalt and lithium.^1,2^ The fluoride-ion battery (FIB) is a promising post-lithium chemistry that has the potential to satisfy both the energy density and sustainability requirements. In conversion-type FIBs, the electrodes undergo multielectron reactions, and charge neutrality is maintained by shuttling a monoanionic charge carrier (F^–^) through the electrolyte.^3^ High theoretical energy densities—on the order of ∼600 Wh kg^–1^—could be obtained owing to the high oxidative stability of F^–^ ions, which enables the use of high-voltage redox pairs.^1^ Additionally, the smaller charge density of the fluoride ion compared to that of traditional cationic charge carriers should provide favorable electrolyte transport properties.^4^ Moreover, the global production of fluorides is over 60 times larger than that of lithium with a large and well-established supply chain.^5,6^

The advancement of fluoride-ion batteries, however, has been hindered by several obstacles. One of these is the lack of a realistic and reproducible testing setup.^7^ Flooded cells (also known as beaker cells) are commonly used to test the electrochemical properties of liquid electrolyte FIBs. However, they impede the realistic assessment of performance given the large excess of electrolyte, the absence of a separator and, in many cases, the use of Pt as the counter electrode, which inevitably results in electrolyte decomposition during cycling.^7−10^

The use of more realistic form-factors such as coin and pouch cells has been hindered by a number of problems including the lack of a reliable counter electrode, equivalent to Li metal in lithium-ion batteries (LIBs). Such an electrode would allow for a more rapid, reproducible, and representative investigation of electrochemical performance, reaction mechanisms, and degradation pathways, for both state-of-the-art and novel FIB materials.

Yaokawa et al. pointed out the problems associated with the use of flooded cells and used a two-electrode setup to cycle BiF_3_ vs Pb. However, their cell exhibited low discharge capacity (110 mAh g^–1^ on first discharge) and poor cycling (∼0 mAh g^–1^ on fifth cycle), partly because of the poor performance of the Pb counter electrode.^7^ Other studies with two-electrode cells have also resulted in poor performance.^11−13^

To design a stable counter electrode, Nowroozi et al. proposed the use of intercalation compounds, particularly La_2_CoO_4_, because of the lower volume changes during cycling (compared to those of conversion-type electrodes), which might provide a more reliable performance. However, the presence of an unknown decomposition reaction in the first cycle, the laborious synthesis procedure of La_2_CoO_4_, and its poor cyclability make it an unsuitable option.^14^ Most of these problems do not only apply to La_2_CoO_4_ but also to intercalation compounds in general.^15−17^

A good counter electrode should exhibit a stable potential during cycling so that any variations in voltage can be attributed to the working electrode. Additionally, it should provide a readily accessible reservoir of F^–^ ions to compensate for irreversible losses caused by the formation of solid electrolyte interphases or the decomposition of the electrolyte. Finally, the counter electrode should exhibit (electro)chemical stability toward F^–^ in the electrolyte.

In this work, we report a novel method to produce reliable and chemically stable Pb-PbF_2_ electrodes featuring a dry-process in the presence of a polytetrafluoroethylene (PTFE) binder. We then introduce a novel liquid electrolyte consisting of highly concentrated tetramethylammonium fluoride in methanol, with propionitrile as the diluent, to demonstrate their suitability as counter electrodes in a two-electrode coin cell setup. Finally, by combining the counter electrode and the electrolyte, we report the best capacity retention for BiF_3_ vs Pb-PbF_2_ full cells to date.

The PbF_2_/Pb redox couple showcases a range of advantages that led to their extensive investigation as active materials in FIBs. These attributes also make them highly suitable for use as a counter electrode. First, the low melting point of Pb (327.5 °C), which facilitates metal crystallization, and the higher ionic conductivity of PbF_2_ at room temperature (10^–7^–10^–9^ S cm^–1^) compared to other fluorides enable excellent conversion yields.^14,18−20^

Additionally, the redox potential of PbF_2_/Pb (−0.15 V vs SHE) falls well within the electrochemical stability window of most FIBs electrolytes. Nevertheless, the cycling behavior of Pb-PbF_2_ electrodes reported thus far is incompatible with their potential merit as stable and reliable counter electrodes, possibly due to the limited focus on their microstructural design.^9,21,22^

A uniform and well dispersed mixture of Pb and PbF_2_ is required to achieve a stable electrode potential, reduce the overpotential during cycling, and provide a reservoir of F-ions.^14^ To achieve full conversion at high current densities, the particle sizes of Pb and PbF_2_ should be minimized.^23^ To accomplish this, PbF_2_ was ball milled until the average particle size was reduced from ∼6 μm to ∼500 nm (Figure 1a,b). The ball-milled PbF_2_ was then annealed at 350 °C to convert the orthorhombic α-crystal structure (10^–8^–10^–9^ S cm^–1^) into the more ionically conductive cubic β-structure (10^–7^–10^–8^ S cm^–1^) (Figure 1a,c).^19^ The average particle size increase during heat treatment due to particle sintering (from ∼500 nm to ∼1.5 μm) is more than compensated by the 1 order of magnitude increase in ionic conductivity (Figure 4c, Figure S1).^19^ Unfortunately, the order of the ball milling and annealing steps cannot be reversed, since the pressure exerted on the particles during the ball milling step converts the β-phase back to the α-structure (Figure 1c).^19^ The minimum particle size of commercially available Pb powder is ∼45 μm, which unfortunately cannot be reduced by ball milling due to the ductility of Pb. Thermal decomposition of Pb(C_2_H_3_O_2_)_2_ was instead used to obtain Pb particles with an average size of 2 μm (Figure 1d,e).^24^

**Figure 1:**
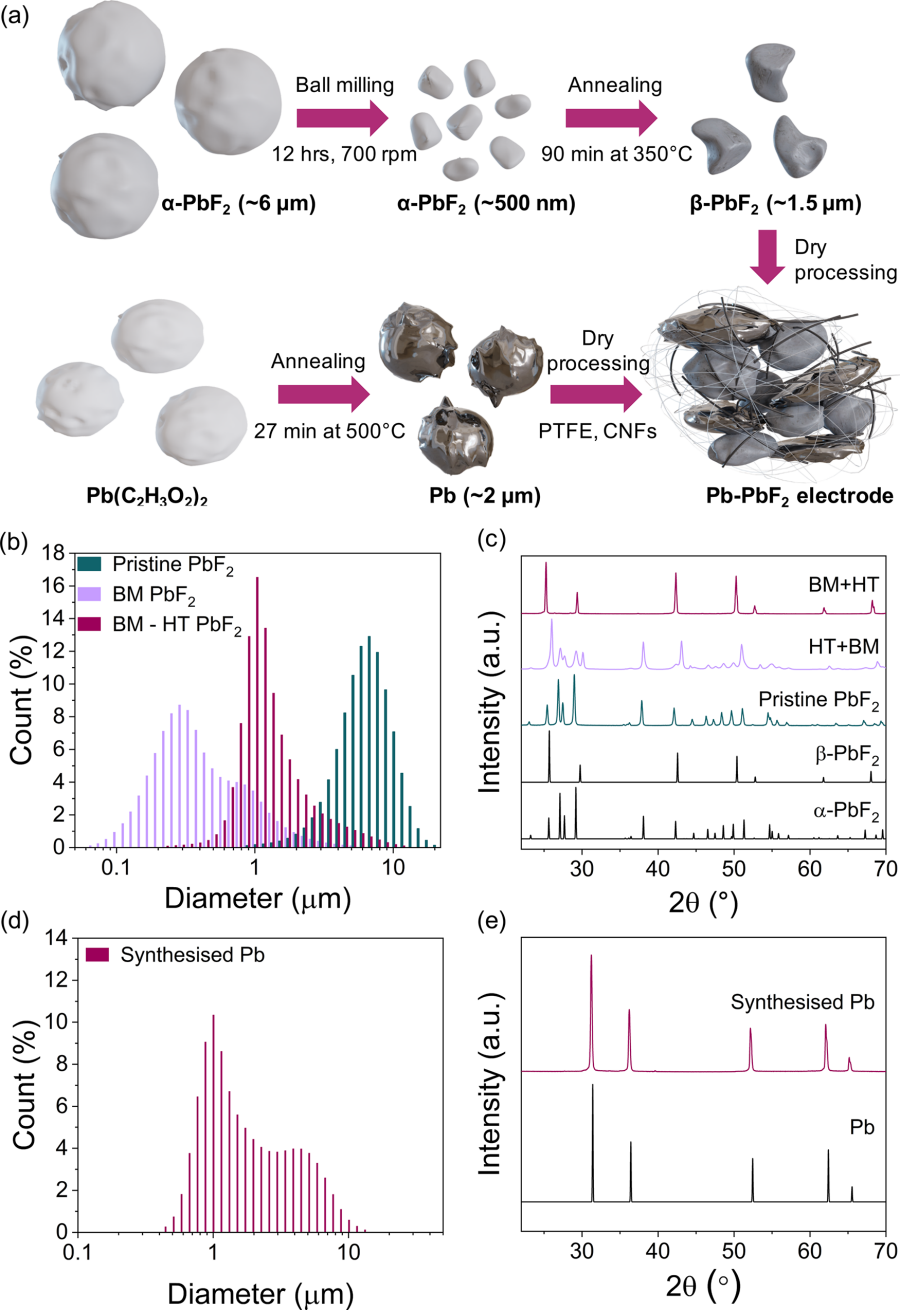
Manufacturing process of Pb-PbF_2_ electrodes. (a) Diagram summarizing the steps in the fabrication of Pb-PbF_2_ electrodes. (b) Particle size distribution for pristine PbF_2_, ball milled (BM) PbF_2_, and final PbF_2_ powder which has been ball milled (BM) and then heat treated (HT). (c) XRD of pristine PbF_2_, PbF_2_ that was first ball milled and then heat treated (BM+HT), and PbF_2_ that was first heat treated and then ball milled (HT+BM). (d) Particle size distribution and (e) XRD pattern of synthesized Pb powder. The black patterns at the bottom of (c) and (e) represent the standards taken from the ICSD database.

PTFE was chosen as the binder because of its chemical compatibility with F-ions.^25^ On the contrary, polyvinylidene fluoride (PVDF), the standard material used as the binder for FIBs, is known to degrade in contact with F-ions, undergoing dehydrofluorination to form HF, C=C bonds, or cross-links.^9,26,27^ To fabricate the Pb-PbF_2_ electrodes with PTFE as the binder, the dry casting manufacturing procedure rather than slurry casting is required, as PTFE is not soluble in common solvents at room temperature.^25^ This method eliminates the need for a solvent evaporation step, leading to time and cost savings.^28^

Pb and PbF_2_ powders were incorporated into the dry casting (Figure 1a) where PTFE fibrils created a 3-D network capable of holding the active material and the carbon nanofibers together (Figure 2a).^29^ As for conductive carbon, carbon nanofibers were chosen due to their ability to interweave with the PTFE fibrils and the metal fluoride particles, providing electronically conductive pathways and structural support.^30^

**Figure 2:**
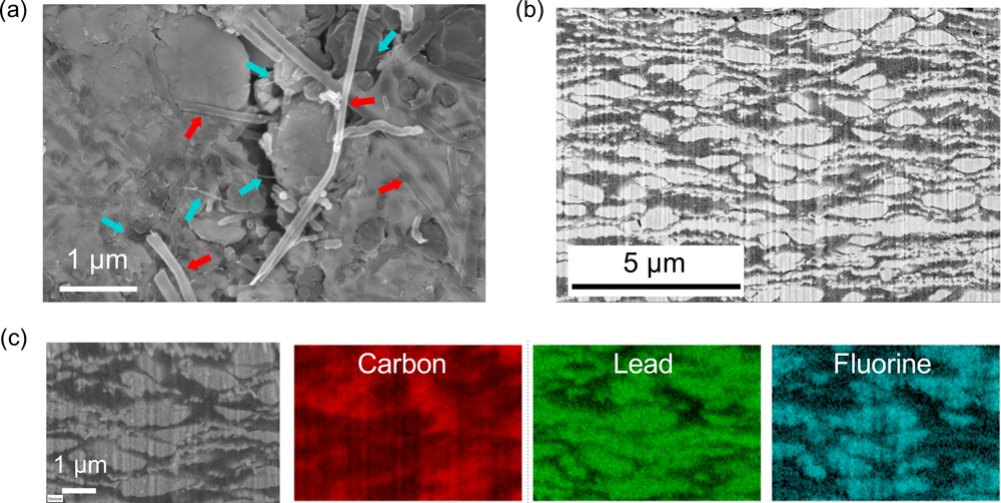
Microstructure of Pb-PbF_2_ electrodes. (a) SEM image of the top surface of a Pb-PbF_2_ electrode with red arrows pointing to the carbon nanofibers while the blue arrows indicate the PTFE fibrils. (b) FIB-SEM cross section with (c) EDS mapping showing how the distribution of Pb, PbF_2_, and carbon is uniform across the thickness of the Pb-PbF_2_ electrode.

The cross section of Pb-PbF_2_ electrodes obtained with focused-ion beam scanning electron microscopy (FIB-SEM) coupled with energy dispersive X-ray spectroscopy (EDS) (Figure 2b,c) demonstrates the uniform distribution of Pb, PbF_2_, and carbon. PbF_2_ particles have an oval shape, whereas Pb particles appear very elongated as a result of the pressure applied during the dry processing and the high ductility of Pb. The morphology of Pb appears to be responsible for the electrode’s low porosity, potentially hindering complete wetting with viscous electrolytes. Both Pb and PbF_2_ are well dispersed in the carbon matrix.

The suitability of Pb-PbF_2_ as a counter electrode in coin cells was validated by using a novel liquid electrolyte. Designing liquid electrolytes for FIBs is challenging owing to the limited solubility of fluoride salts in the aprotic organic solvents commonly used in Li-ion batteries. Protic organic solvents are particularly effective at dissolving fluoride salts thanks to their ability to form hydrogen bonds, but the very same hydrogen limits their cathodic (electro)chemical stability. In our previous work we have demonstrated the benefits of using solvent-in-salt electrolytes in FIBs to expand the electrochemical stability window of protic solvents by suppressing the number of free solvent molecules as well as minimizing HF formation.^31^ This strategy, however, requires an impractically large amount of salt and results in high viscosity, which can negatively impact the transport and wetting properties. The use of a diluent (propionitrile, PN) was therefore demonstrated herein, for the first time in an FIB, to drastically reduce the quantity of salt needed and to improve the transport properties of a highly concentrated solution (10 m) of tetramethylammonium fluoride (TMAF) in methanol (MeOH). Propionitrile was selected because of its wide electrochemical stability window (>5 V), low viscosity (0.399 mPa.s at 25 °C), miscibility with alcohols, and high boiling point (97 °C).^32,33^ Additionally, propionitrile does not dissolve TMAF (Figure S4) and is chemically stable toward fluoride ions.^34^ A ratio of 5 wt % MeOH/95 wt % PN resulting in a TMAF concentration of 0.5 m was chosen to investigate how a high diluent concentration affects the electrolyte properties.

A combination of spectroscopic and computational characterization methods was employed to investigate the properties of the liquid electrolyte, in particular the solvation of fluoride ions and the role of the diluent. ^1^H NMR shows a 2.3 ppm shift to higher frequencies in the peak corresponding to the -OH proton between highly concentrated TMAF in MeOH and pure MeOH (Figure 3a). This indicates that fluoride ions are preferentially solvated by methanol via OH··· F^–^ hydrogen bonding (Figure 3b). The relative bonding distance between the hydroxylic proton in methanol and the fluoride ions was calculated to be 1.58 Å by Monte Carlo simulations (Figure 3b, S13), in agreement with previously calculated bond distances.^35^ Within the first solvation shell, TMA^+^ counterions are found at a distance of 2.55 Å from F^–^, due to the strong Coulombic interactions and the tendency to form ion-pairs and aggregates in highly concentrated solutions (Figure 3b). This model indicates an average of approximately two methanol molecules (∼2.3 ± 0.1) interacting with a fluoride ion, and this number decreases to 0.5 ± 0.1 when propionitrile is added, due to the competing interaction arising between PN and MeOH and the large excess of the former (Figure 3b,c). As a result of the insolubility of TMAF in PN, propionitrile acts as a diluent by interacting with F-ions via weaker van der Waals forces, which do not alter the distance between F^–^ and MeOH, and reducing the viscosity of the highly concentrated electrolyte (Figure 3c, Figure S4).^36^ In the diluted electrolyte, the reduced viscosity coupled with the lower number of coordinated MeOH molecules resulted in a 1 order of magnitude increase in the fluoride diffusion coefficient (1.4 × 10^–9^ m^2^ s^–1^) compared to the highly concentrated electrolyte (2.05 × 10^–10^ m^2^ s^–1^). Meanwhile, the ionic conductivity was still reasonably high at 7 mS cm^–1^, decreasing from 28 mS cm^–1^ in the 10 m electrolyte due to the considerably lower number of charge carriers in the diluted electrolyte (Figure 3b,c). In addition to improving F-ion transport and decreasing the amount of salt required, the diluted electrolyte maintains the advantage of the highly concentrated electrolyte given the unaffected electrochemical stability window upon addition of PN (Figure 3d).

**Figure 3:**
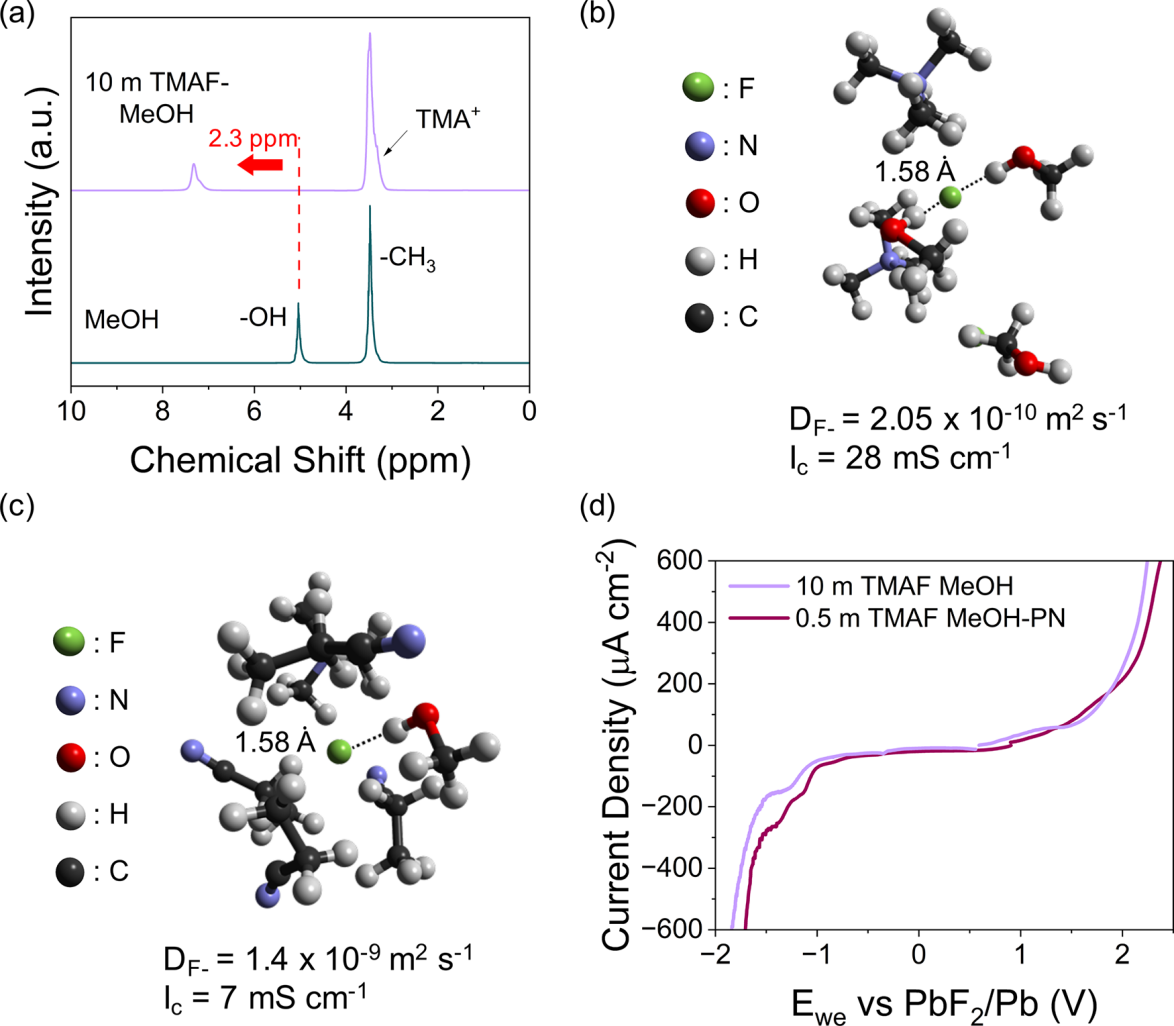
Electrolyte characterization. (a) ^1^H NMR showing a shift to higher frequencies in the proton peak of the -OH group in 10 m TMAF in MeOH compared to pure MeOH. (b and c) Snapshot of Monte Carlo simulation boxes showing F^–^ solvated by (b) methanol molecules in the 10 m solution and (c) methanol and propionitrile molecules in the 0.5 TMAF-MeOH-PN ternary solution. Their respective diffusion coefficients (D_F-_) and ionic conductivities (I_c_) are also reported. In both electrolytes the distance between fluoride ions and the proton of MeOH is 1.58 Å. (d) Electrochemical stability window of 10 m TMAF in MeOH and 0.5 m TMAF in MeOH-PN measured at a scan rate of 1 mV s^–1^.

The electrochemical behavior of the Pb-PbF_2_ electrodes was then tested in symmetric coin cells via galvanostatic cycling and ex-situ XRD. A PTFE-based separator was used to ensure chemical stability. Since the cells were assembled in a 50% state of charge, the maximum capacity accessible on the first discharge is half the theoretical capacity (109 mAh g^–1^). Starting from the first charge, the maximum achievable capacity corresponds to the theoretical capacity of PbF_2_ (218 mAh g^–1^). On the first discharge, 106 out of the 109 mAh g^–1^ is accessed (Figure 4a), suggesting that almost all of the active material undergoes the conversion reaction. On the first charge, the capacity reaches 211 out of the 218 mAh g^–1^ and then decreases to 202 mAh g^–1^ on the second discharge (Figure 4a,b).

**Figure 4:**
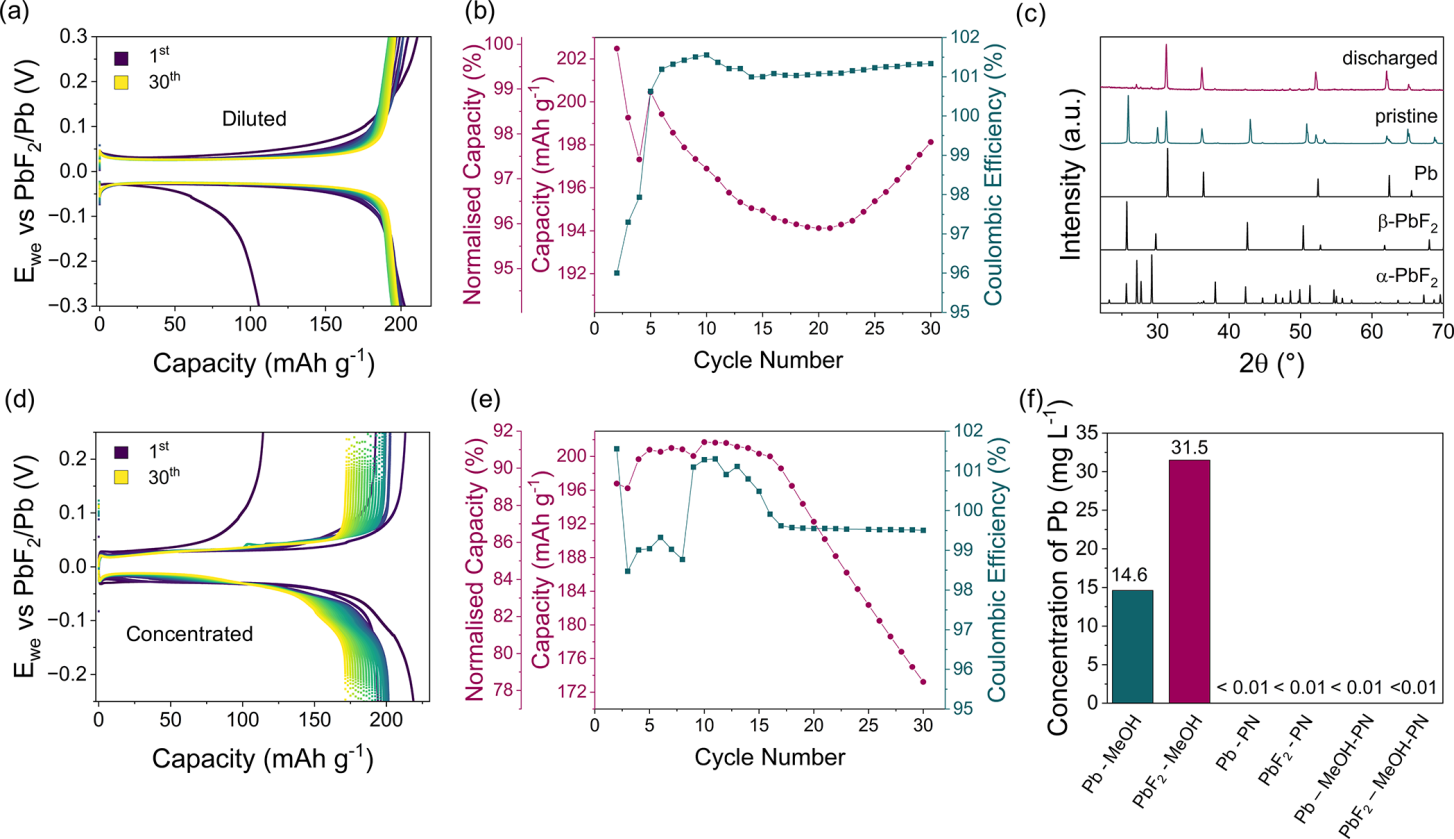
Electrochemical performance of Pb-PbF_2_. (a) GCPL plot showing the cycling of a Pb-PbF_2_ symmetric cell with 0.5 m TMAF in MeOH-PN at a C/20 rate (10.9 mA g_PbF2_^–1^). (b) Capacity, normalized capacity, and Coulombic efficiency as a function of cycle number for the Pb-PbF_2_ symmetric cell. (c) XRD demonstrating full conversion to Pb metal after the first discharge. (d) GCPL plot showing the cycling of a Pb-PbF_2_ symmetric cell in the highly concentrated electrolyte at a C/20 rate (10.9 mA g_PbF2_^–1^). (e) Capacity, normalized capacity, and Coulombic efficiency as a function of cycle number for the Pb-PbF_2_ symmetric cell. (f) ICP showing Pb and PbF_2_ dissolution in MeOH but not in PN or a mixture of the two. The ratio in the MeOH-PN mixture is 5 wt % methanol and 95 wt % propionitrile as in the diluted electrolyte. The black spectra at the bottom of (c) represent the standards taken from the ICSD database.

The XRD at the end of the first discharge confirms that the obtained capacity arises from the conversion to the Pb metal (Figure 4c). The cell retained 97.8% of its original capacity after the 30th cycle with a low overpotential of 30 mV on both charge and discharge (Figure 4a,b), outperforming previously reported Pb-PbF_2_ electrodes, including in flooded cells.^9,14,21,22^ The capacity retention obtained with the diluted electrolyte is higher than that achieved with the highly concentrated electrolyte (78.5% at the 30th cycle) (Figure 4d,e), possibly due to the lower active material dissolution. Inductively coupled plasma spectroscopy (ICP) results show that PbF_2_ and Pb are soluble in methanol up to 31.5 and 14.6 ppm, respectively, whereas neither of the two is soluble in PN (<0.01 ppm) (Figure 4f). Additionally, the 5 wt % MeOH in the diluted electrolyte is not sufficient to cause any detectable dissolution, allowing for improved cycling performance in the diluted electrolyte compared to the highly concentrated one. The presence of a reliable counter electrode and the realistic cell design allowed meaningful measurements of Coulombic efficiency, rarely reported for FIBs.

The Coulombic efficiency of the cell with the diluted electrolyte started at 96% in the first cycle and, from the 15th cycle, plateaued at 101%. (Figure 4b). This could be indicative of a parasitic reaction with the electrolyte.

To prove the feasibility of Pb-PbF_2_ as a counter electrode in coin cells, it was tested against BiF_3_ working electrodes. On the first discharge, a capacity of 284 mAh g^–1^ out of the theoretical 302 mAh g^–1^ is achieved, suggesting that most of the BiF_3_ is converted to Bi metal (Figure 5a,c). Upon recharge, a considerably lower capacity of 178 mAh g^–1^ is reached, indicating that not all of the Bi metal is converted back to BiF_3_ (Figure 5a,b, Figure S12). Despite not achieving full conversion, BiF_3_ cycles reversibly with a capacity retention of 173 mAh g^–1^ (61%) after 10 cycles, making this cycling performance the best for BiF_3_ in a FIB (Table S1). Similar to the Pb-PbF_2_ symmetric cell, the Coulombic efficiency is over 100% and appears to plateau at 101% on the ninth cycle, suggesting some minor degradation processes are taking place (Figure 5b). The asymmetry between the charge and discharge profiles has already been observed by Yaokawa et al. and Okazaki et al., and a detailed mechanistic study would be required to elucidate its origin.^7,11^ The coin cell setup presented in this work could offer an ideal configuration for conducting such an investigation through in-situ XRD.

**Figure 5:**
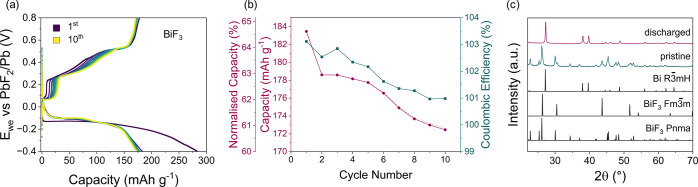
Electrochemical performance of BiF_3_. (a) Cycling of BiF_3_ vs the Pb-PbF_2_ counter electrode at a rate of C/20 (15.1 mA g_BiF3_^–1^). (b) Corresponding capacity retention and Coulombic efficiency as a function of cycle number. (c) XRD demonstrating the conversion from BiF_3_ to Bi metal after the first discharge. The black spectra at the bottom of (c) represent the standards taken from the ICSD database.

In conclusion, we present a novel manufacturing method for Pb-PbF_2_ electrodes using a dry-casting process using PTFE as the binder and validate their use as counter electrodes in two-electrode coin cells using a novel diluted liquid electrolyte. With this electrolyte, we report, for the first time in FIBs, the use of a diluent (propionitrile) to improve the transport properties and reduce the amount of salt required by a highly concentrated electrolyte (tetramethylammonium fluoride in methanol), while retaining the wider electrochemical stability window. Finally, we demonstrate the suitability of Pb-Pb_2_ as counter electrodes in coin cells by cycling versus BiF_3_ electrodes, prepared via the same dry-casting process. The capacity retention obtained translates to the best electrochemical performance for this system to date. We believe that the introduction of a reliable counter electrode for a practical and accessible cell configuration (like coin cells) is a critical step to the advancement of FIBs, as it will empower a more streamlined development and understanding of novel active materials and the investigation of degradation mechanisms. We hope that this work, despite requiring further optimization, could be of inspiration to the FIB community.

## Supporting Information Available

Experimental methods for electrode manufacturing and characterization, electrolyte synthesis and testing, and cell assembly; supplemental figures and tables including SEM images of PbF_2_ after milling and heat treatment, XRD patterns during cycling, ESW of TMAF in MeOH, ^1^H NMR of TMAF in PN, cycling stability and dQ/dV plots for the counter electrode, electrochemical impedance spectroscopy characterization and fitting, cycling behavior of BiF_3_ vs Pb-PbF_2_, Lennard-Jones parameters, and radial partial distribution functions (PDF)
