# Supplementary material for: Advancing Fluoride-Ion Batteries with a Pb-PbF2 Counter Electrode and a Diluted Liquid Electrolyte
Source: ACS Energy Lett. 2023 Dec 8;9(1):85–92. doi: 10.1021/acsenergylett.3c02228 (PMC10789089; doi:10.1021/acsenergylett.3c02228)
Supplement: Supplementary file 1 — nz3c02228_si_001.pdf [file nz3c02228_si_001.pdf]

SI - Advancing fluoride-ion batteries with a Pb-PbF_2 counter electrode and a diluted liquid electrolyte

Giulia Galatolo[1], Omar M. Alshangiti[1], Camilla Di Mino[1,2], Guillaume Matthews[1,2], Albert W. Xiao[1], Gregory J. Rees[1], Maximilian Schart[1], Yvonne A. Chart[1,2], Lorenz F. Olbrich[1], Mauro Pasta[1✉]

1. Department of Materials, University of Oxford, Oxford, OX1 3PH, United Kingdom.

2. The Faraday Institution, Harwell Campus, Quad One, Becquerel Avenue, Didcot OX11 0RA, United Kingdom

[1✉] Correspondence: mauro.pasta@materials.ox.ac.uk

Methods

S0.1. Electrode Manufacturing

Lead fluoride (PbF_2 99.99%) and lead acetate trihydrate ($Pb(C_2H_3O_2)_2$ 99%) were purchased from Sigma Aldrich. PbF_2 was wet-ball milled with octadecene and oleic acid for 12 hours at 700 rpm with 7-minute long pauses every ten minutes. It was then heated up to 350°C in a Ar-filled glovebox (O_2 and H_2O < 0.1 ppm) for 90 minutes to transform the α-PbF_2 to β-PbF_2. Pb powder was produced by heating $Pb(C_2H_3O_2)_2$ at 500°C for 27 minutes in an Ar-filled glovebox. Carbon nanofibres (CNFs) and polytetrafluoroethylene (PTFE) powder (675 μm particle size) were purchased from Sigma Aldrich and Goodfellow, respectively, and dried under vacuum at 100°C overnight. Pb (45 wt%), PbF_2 (45 wt%), CNFs (9 wt%) were mixed with a mortar and pestle until a uniform colour could be observed. PTFE (1 wt%) was then added to the mortar and mixed with the other components until uniformly dispersed. Pressure was then applied with the pestle to initiate the fibrilisation process. The powders started to compact into flakes suggesting that 3-D network had started to form. Eventually, all the flakes merged together to form a unique film. The film was inserted between two stainless steel sheets for the calendaring step. The high and uniform pressure applied by the rolling machine allowed to increase the mechanical strength of the film by continuing the fibrilisation process as well as tune the thickness of the freestanding casting. The film was calendared until the desired thickness of 80 μm was reached. 5-mm diameter electrodes were then punched out from the film.

BiF_3 (99%) was purchased from Sigma Aldrich and ball milled using the same conditions as PbF_2. BiF_3 electrodes were made using the dry processing previously described, using a ratio of 70 wt% active material, 20 wt% carbon and 10 wt% PTFE. The Pb-PbF_2 counter electrode used to cycle BiF_3 has a 65(Pb):15(PbF_2):10(C):10(PTFE) wt% ratio. An excess of Pb-PbF_2 corresponding to a N:P ratio of 2.2 was used.

S0.2. Electrode Characterisation

The particle size distribution was measured using dynamic light scattering (DLS) with a Partica LA-960V2 analyser (HORIBA).

X-ray diffractometry (XRD, Rigaku Miniflex 600) was carried out with a Cu Kα radiation source in a nitrogen-filled glovebox with O_2 and H_2O contents less than 1 ppm.

The SEM analysis was performed in a Zeiss Merlin FEG-SEM at an accelerating voltage of 10 kV. In addition, the samples were coated with 4 nm of platinum using a Leica EM ACE200 coating system, in order to avoid local charging and improve image resolution.

Cross-sectional SEM images were prepared using a Helios G4 PFIB CXE Dual Beam (Thermo Scientific). After depositing a protective layer of organometallic Pt, all milling was done at 30kV and the samples were polished to 60nA to balance time taken and quality of the cross-section. SEM images were taken with 5kV 0.1nA and EDS was run with 5kV 1.6nA to obtain a more surface-sensitive signal.

S0.3. Electrolyte Synthesis

Tetramethylammonium fluoride was synthesised adapting the procedure reported by Christe et al.[1] Tetramethylammonium tetrafluoroborate trihydrate ($TMABF_4$) (97%) and potassium fluoride (KF) (99%) were both purchased from Sigma Aldrich. Methanol (>99.9%) and propionitrile (>99.9%) were purchased from Sigma Aldrigh and dried under 3 Å molecular sieves to reduce the water content below 15 ppm. A concentration of 10 m was used for the highly concentrated TMAF in MeOH. The 0.5 m TMAF in MeOH-PN was produced by maintaining the ratio between between TMAF and MeOH constant at 10 m and adjusting the amount of PN to reach the final 0.5 m concentration.

S0.4. Electrolyte Characterization

The ionic conductivity was measured using electrochemical impedance spectroscopy (EIS). Measurements were acquired between 1 MHz and 100 mHz using a custom-made PEEK cell having stainless steel blocking electrodes. The cell was rested for 30 minutes before running the measurement to ensure the system had reached the desired temperature of 30°C. The ionic conductivity was calculated using the linear fit of the Nyquist plot.

The diffusion coefficients was measured at 30°C using pulsed field gradient (PFG) NMR, with measurements completed at 9.45 T ($\nu_0(^{19}F) = 376.51$) on Bruker Avance III HD spectrometer using a 5 mm single-axis diffusion probe with exchangeable ceramic heads. A stimulated echo pulse sequence was utilized for all PFG experiments with effective gradient pulse durations (δ) ranging between 1 to 1.5 ms, and a diffusion time (Δ) of 10-30 ms, with the gradient amplitude varying between 0.1 and 24 T/m in 36 steps. All samples were sealed

in a J-Young air-tight NMR tube, temperature stabilized to 303.1 K, and a 1-second recycle delay was used throughout. All data was fitted to the Stejskal-Tanner equation:

$$f(x) = I_0 e^{-\gamma^2 g^2 \delta^2 \frac{(\Delta-\delta)}{3} D} \tag{S1}$$

An R^2 value of 0.999, errors of $< 1\%$ and normal distribution of the residuals (>0.5 Shapiro Wilko score) were observed for all experiments.

The electrochemical stability window was measured by means of linear scanning voltammetry (LSV) using a PTFE custom-made cell. A 3-mm diameter glassy carbon pin and a Pt wire were used as the working (WE) and counter electrodes (CE), respectively. A Pb-PbF_2 dry casting electrode was used as the pseudo-reference electrode (RE). A scanning rate of 1 mV/s was used.

A Perkin-Elmer Optima 8000 ICP-OES instrument was used to quantify the dissolution of Pb and PbF_2 in methanol and propionitrile. The extent of Pb and PbF_2 dissolution was measured separately in methanol, propionitrile and a 5 wt% MeOH- 95 wt% PN solution. To do so, ~3 mg of Pb or PbF_2 were mixed with ~3 ml of solvent. After 20 hours, the solutions were centrifuged, and the supernatant filtered with 100-nm PTFE filters. Equal volumes of sample and a Mn (100 ppm) ICP standard solution were mixed and diluted by a factor of 100 to 300 depending on the sample. The dilution was done with a 2% HNO_3 solution in ultrapure water.

S0.5. Cell Assembly and Testing

CR2032 coin cells (20 mm diameter, 3.2 mm thickness) with stainless steel spacers (15.5 mm diameter, 0.5 mm thickness) (PI-KEM) were used. Omnipore hydrophilic PTFE membranes (65-μm thick, 0.1 μm pore size) were employed as the separators. 2 spacers were used to ensure sufficient pressure inside the coin cell, whereas 2x18-mm diameter PTFE separators were used to prevent short circuit. An electrolyte excess was used. The cells were cycled using a Biologic VMP3 potentiostat.

Supplementary Text

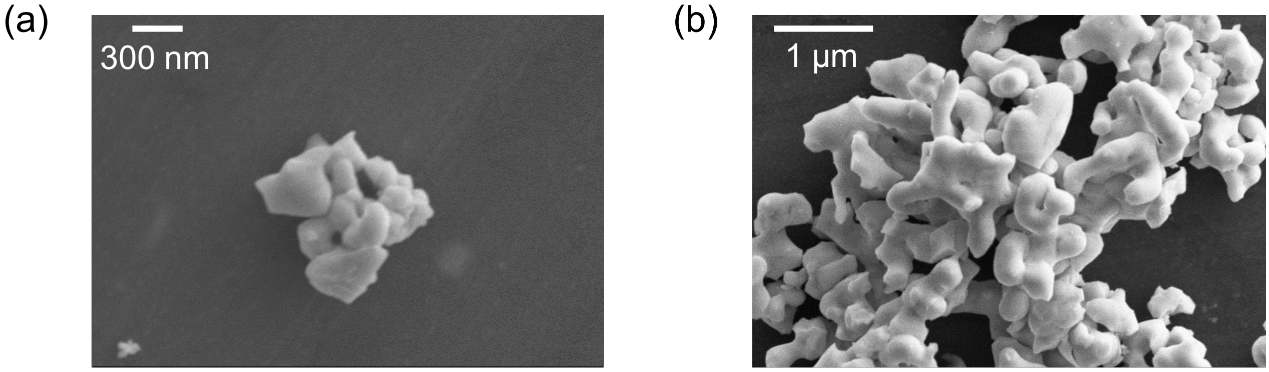

Figure S1: **Size of PbF_2 particles.** (a) PbF_2 particles after ball milling (b) PbF_2 particles after ball milling and heat treatment.

PbF_2 particles after ball milling are around few hundreds nm in size and have roughly spherical shape. After the heat treatment, the particles appear larger, in the range of 1 μm. The elongated shape appears to result from the sintering of different particles together because of the heat treatment.

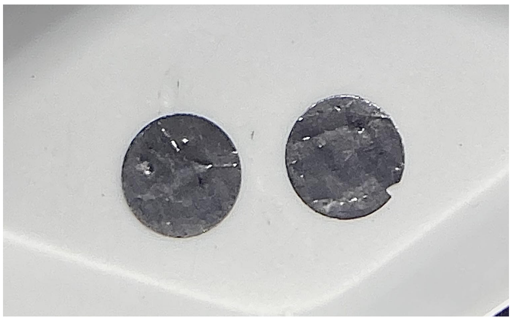

Figure S2: **Pb-PbF_2 electrodes made via dry processing**

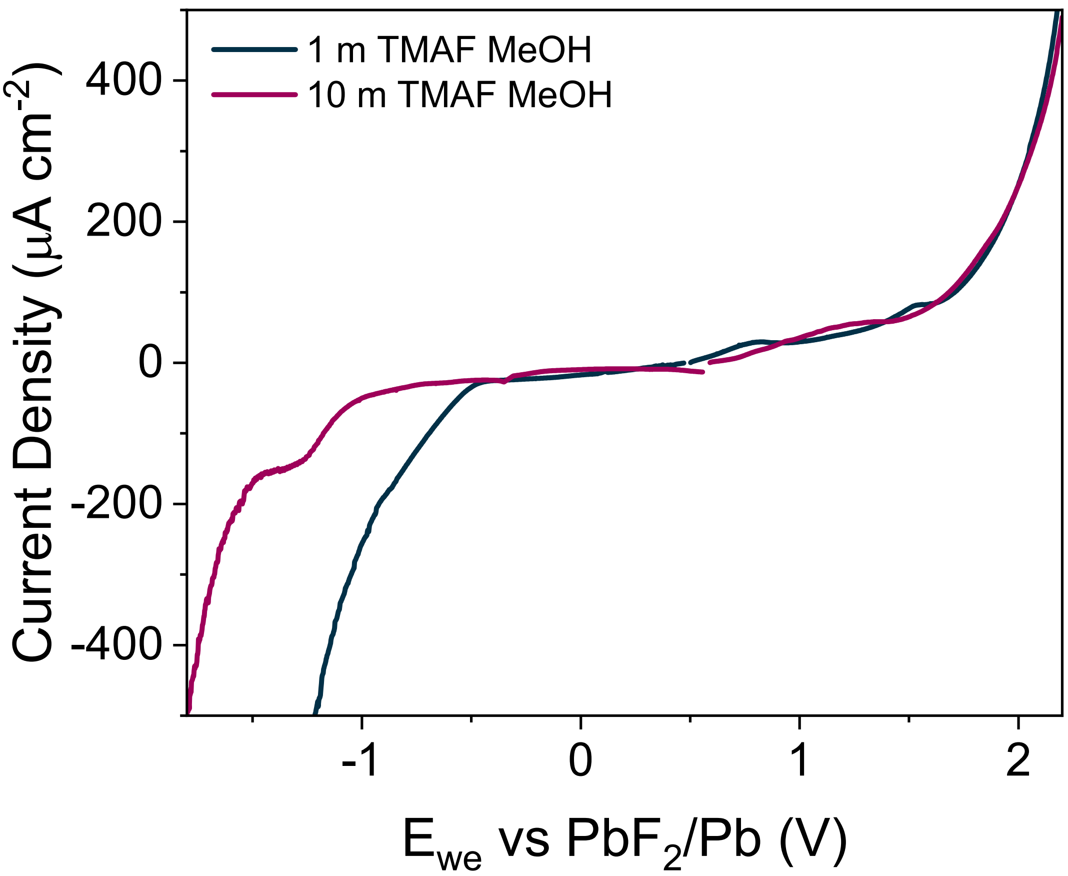

Figure S3: **ESW of TMAF in MeOH.**

By increasing the concentration of TMAF in MeOH from 1 m to 10 m the electrochemcial stability window expands by 0.5 V as previously demonstrated for FIB solvent-in-salt electrolytes.[2]

The shift in the ESW of 10 m TMAF in MeOH towards low potentials arises from the use of a pseudo-reference electrode. The higher concentration of F-ions in solution in the case of 10 m TMAF-MeOH affects the potential of the PbF_2/Pb pseudo-reference electrode, resulting in the observed shift.

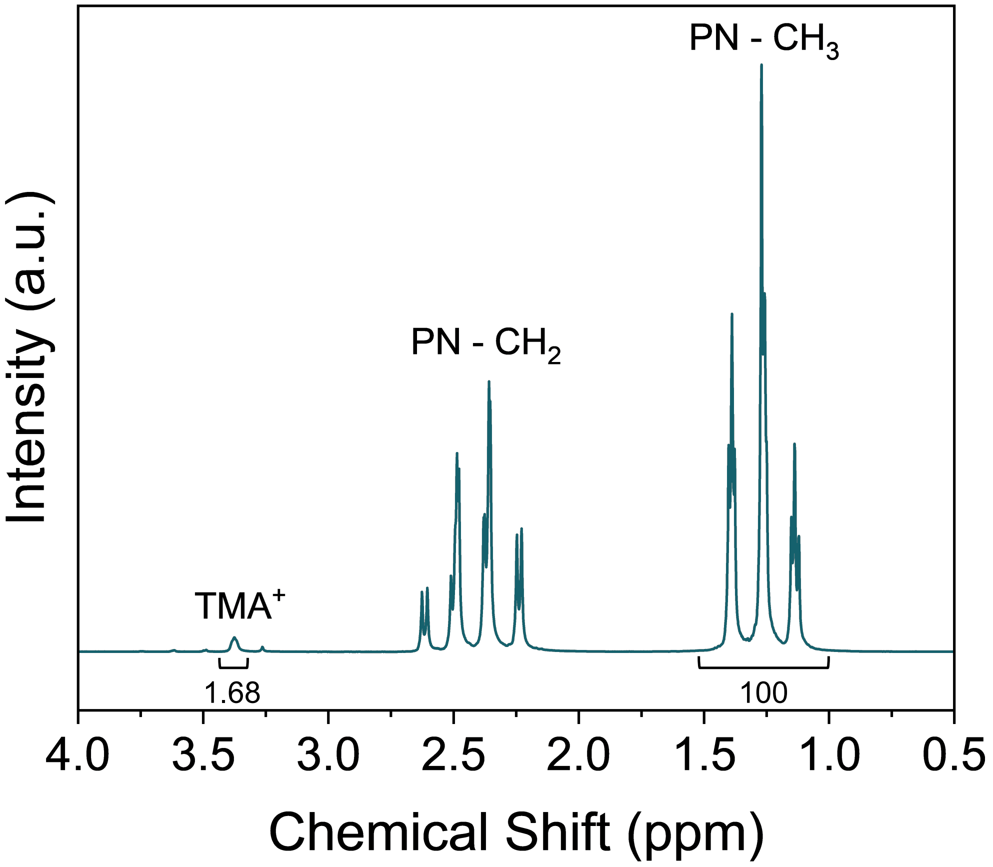

Figure S4: **^1H NMR of TMAF in propionitrile. 1.68 and 100 are the integrals of the peaks of TMA^+ and the methyl group of PN, respectively.**

To test the solubility of TMAF in PN, a supersaturated solution was equilibrated at 25°C and the excess TMAF was filtered out. The integral ratio in ^1H NMR was used to calculate the maximum concentration of TMAF dissolved in PN, to be 0.2 x 10^{-3} m, which makes TMAF practically insoluble in PN.

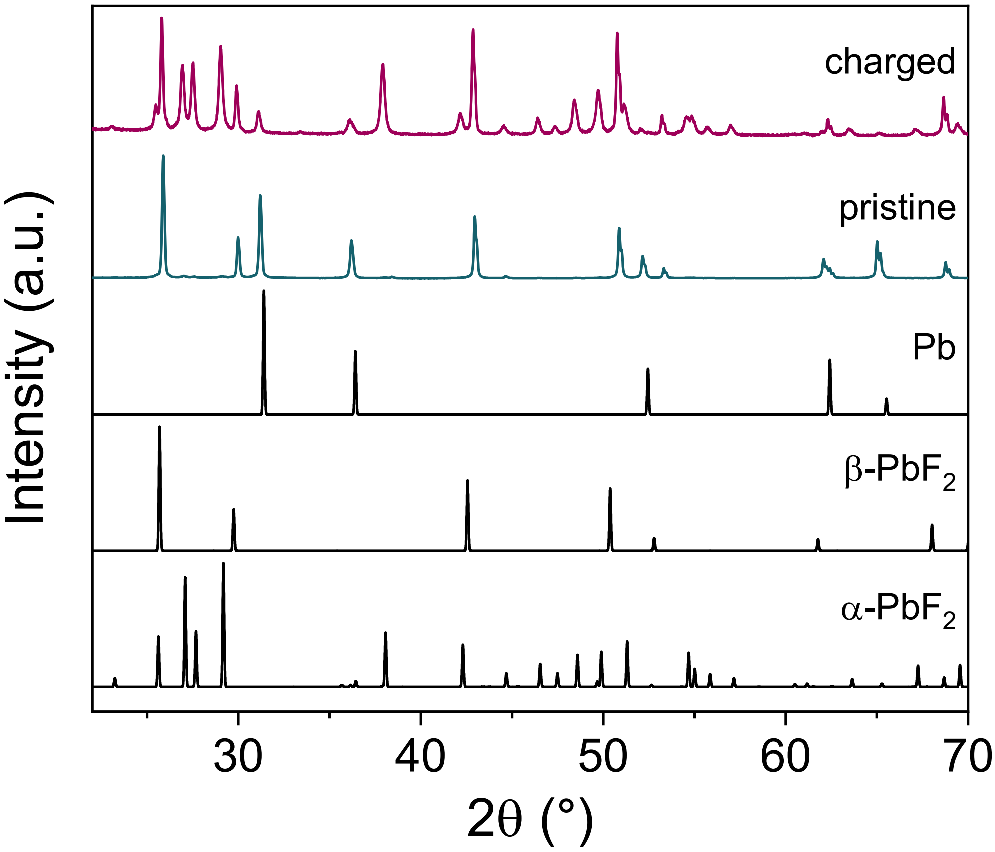

Figure S5: **XRD of charged Pb-PbF$_2$ symmetric cell.**

Upon recharge the β-crystal structure of PbF$_2$ converts to the α-phase, suggesting it is the most kinetically stable under these conditions.[3] In the literature, both the reversible cycling of β-PbF$_2$ and the conversion from β- to α-PbF$_2$, as in this case, have been reported.[3,4] More work is required to understand the underlying causes of this interesting mechanism. The coin cell set-up reported in this study offers the perfect conditions for such an investigation.

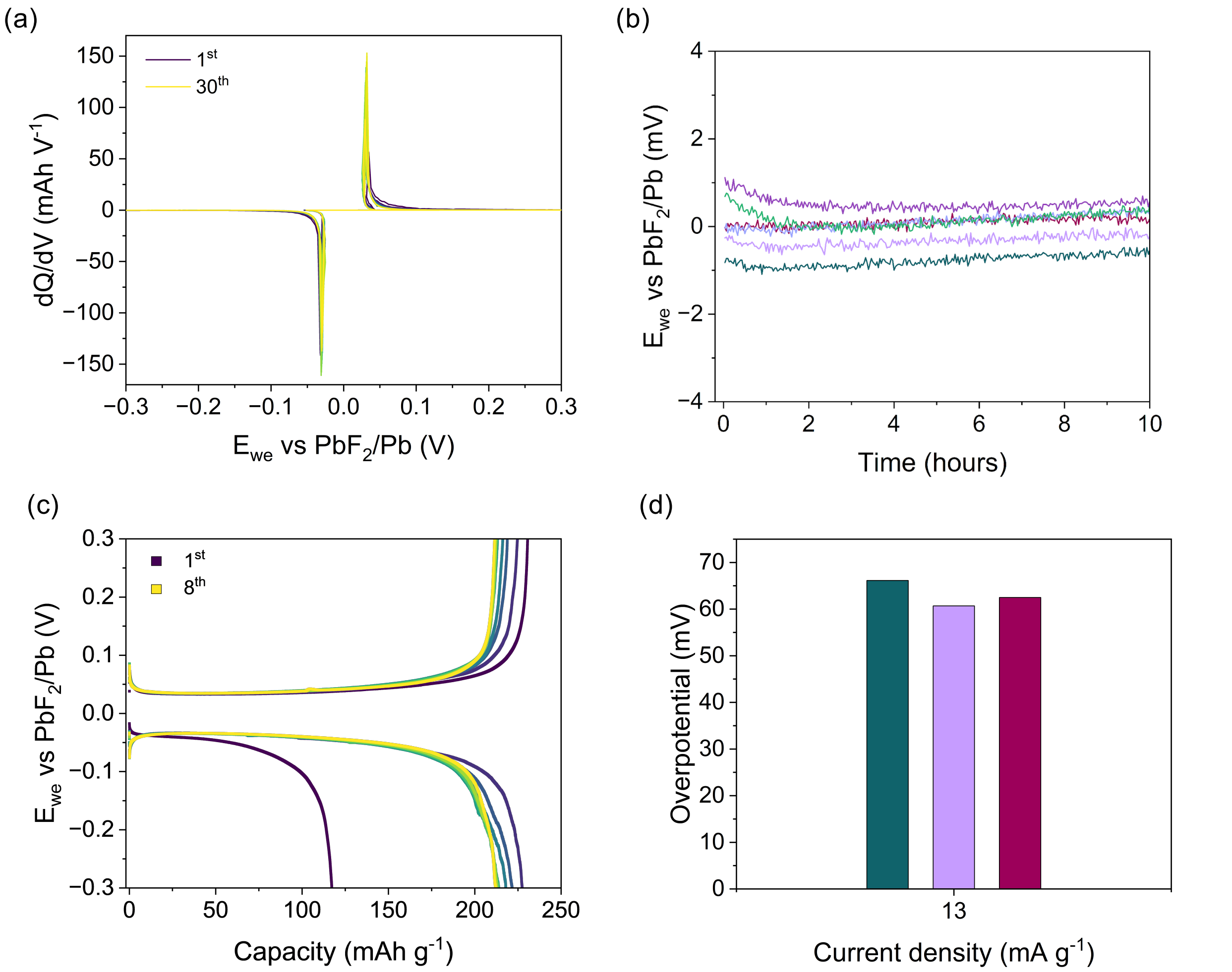

Figure S6: **Stability and reliability of the Pb-PbF_2 counter electrode. (a) dQ/dV plot of a Pb-PbF_2 symmetric cell. The unique sharp peak on both charge and discharge indicates the constant overpotential during cycling. (b) Open circuit potential of six Pb-PbF_2 symmetric cells. The potential for all cells remains constant for at least 10 hours. (c) Cycling behaviour of a second Pb-PbF_2 symmetric cell. (d) Overpotential for three symmetric Pb-PbF_2 cells cycled at a current density of 13 mA/g. The overpotential was measured at 50% SOC. The three cells have very similar overpotentials.**

To further illustrate the stability of the Pb-PbF_2 counter electrode, the dQ/dV plot for the cell cycled in Figure 4a is reported in Figure S6a. This plot displays only one sharp peak (corresponding to the Pb-PbF_2 conversion reaction) on both charge and discharge at ±30 mV indicating the constant potential of the charge and discharge plateaus of the galvanostatic plot. Additionally, the open circuit potential of six Pb-PbF_2 symmetric cells is displayed in Figure S6b to demonstrate how the potential does not change over time and how all the symmetric cells have a cell potential within 1 mV.

To further demonstrate the reliability of the counter electrode, the cycling behaviour of a second Pb-PbF_2 symmetric cell is reported in Figure S6c showing a similar capacity to the one obtained in Figure 4a and a good capacity retention up to the 8th cycle. The overpotential of three symmetric Pb-PbF_2 cells cycled at the same current density of 13 mA g^{-1} is displayed in Figure S6d. The three cells exhibit a consistent overpotential (61, 62.5 and 66 mV).

(a)

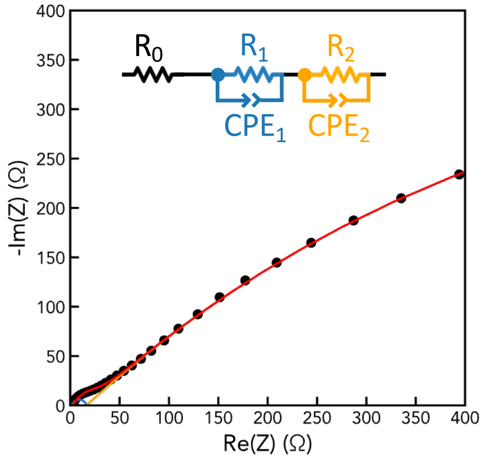

(b)

Fitting Element	Resistance (Ω)	Capacitance (s^{α}/Ω)	α
R^0	1.6	-	-
R^1	14.79	-	-
CPE^1	-	$1.15{\cdot}10^{-4}$	0.85
R^2	1632	-	-
CPE^2	-	$2.19{\cdot}10^{-3}$	0.47

Figure S7: **Origin of overpotential in Pb-PbF_2 symmetric cells. (a) Nyquist plot at 50% state of charge with the insert displayng the corresponding equivalent circuit. (b) Fitting parameters for (a).**

To investigate the origin of the overpotential in the Pb-PbF_2 symmetric cell, an electrochemical impedance spectroscopy investigation was carried out. The Nyquist plot at 50% state of charge is shown in Figure S7a. The data was fitted with the equivalent circuit reported in the insert of Figure S7a and the fitting parameters in Figure S7b. R_0 is the resistance associated with the electrolyte and the electrical connections. The $R_1|CPE_1$ element has a time constant of 1.7 ms which is typical of interfacial processes including charge transfer resistance and solid electrolyte interphases. The $R_2|CPE_2$ has a time constant of 3579 ms consistent with a solid diffusion process. The PbF_2 particles used in this work have an average particle size of 2 μm that with R_2 leads to an ionic conductivity of 3 x 10^{-8} S cm^{-1}, which is in good agreement with the ionic conductivity of PbF_2. The diffusion in PbF_2 therefore seems to be the main source of the overpotential.

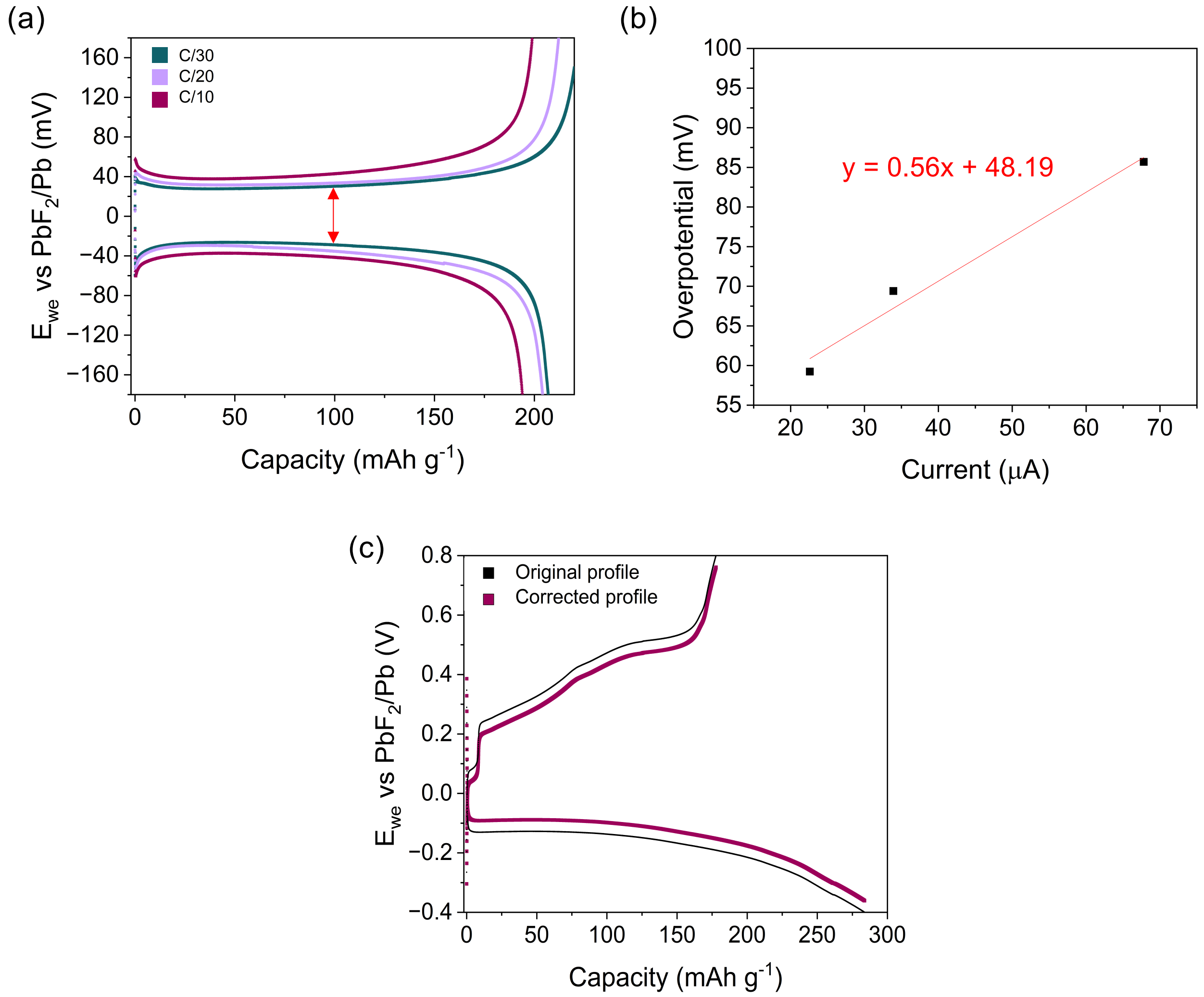

Figure S8: **Pb-PbF_2 overpotential correction. (a) Cycling profile of a symmetric Pb-PbF_2 cell cycled at C/10, C/20 and C/30. (b) Plot of overpotential at 50% SOC versus current for the Pb-PbF_2 cell in (a). The linear fit between the data points enables the interpolation of the overpotential at the current used to cycle the BiF_3|Pb-PbF_2 cell. (c) Cycling profile of BiF_3 versus Pb-PbF_2 counter electrode before (black line) and after (red line) the overpotential correction of the Pb-PbF_2 counter electrode.**

Galvanostatic charge and discharge of a symmetric Pb-PbF_2 cell at different current densities could be used as a method to correct the curves of the working electrode by the overpotential of the Pb-PbF_2 counter electrode. Three current densities C/10 (67.8 μA), C/20 (33.9 μA), C/30 (22.6 μA) (Figure S8a) were selected, whose range include the value used to cycle the BiF_3|Pb-PbF_2 cell (46.3 A). The total overpotential at 50% state of charge (SOC) for each current density was then plotted as a function of the applied current (Figure S8b). The linear fit of the three data points (Figure S8b) was used to interpolate the overpotential of the Pb-PbF_2 cell at the current used to cycle the BiF_3|Pb-PbF_2 cell (74.1 mV). The symmetric cell is composed of two Pb-PbF_2 electrodes and therefore to obtain the contribution of one single counter electrode the total overpotential needs to be halved (37 mV). Figure S8c displays the cycling profile of the BiF_3|Pb-PbF_2 cell before (black line) and after (red line) the overpotential correction.

At the cycling rates used in this work the contribution of the Pb-PbF_2 counter electrode to the total overpotential is quite small. This would be even more true for the slower rates commonly used in the literature. However, if more practical current densities were to be used, this correction would be necessary.

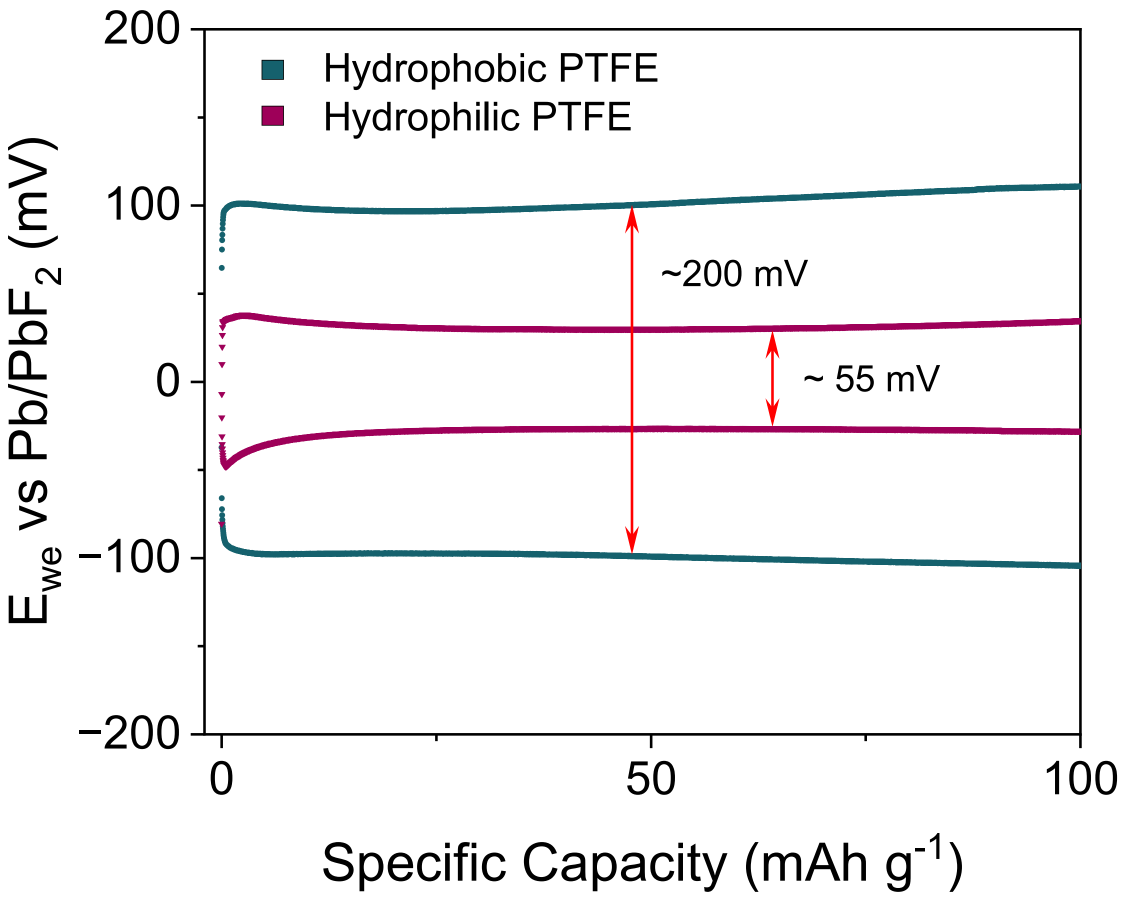

Figure S9: **Effect of separator on the cycling overpotential of symmetric Pb-PbF$_2$ cells.**

The hydrophilic coating on the PTFE separator reduces the overpotential during cycling by 150 mV compared to a standard hydrophobic PTFE separator.

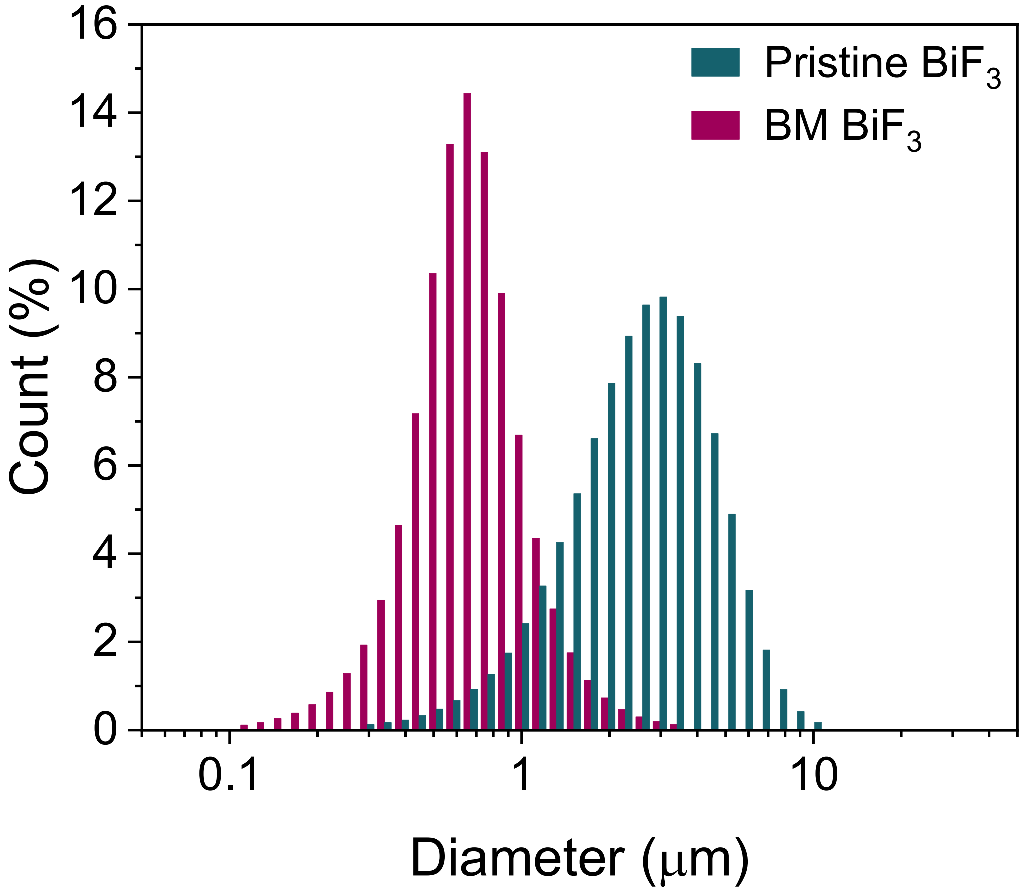

Figure S10: **Particle size distribution of BiF_3 before and after ball milling**

The commercial BiF_3 powder has a median particle size of 2.4 μm, which decreases down to 0.62 μm after 12 hours of ball milling.

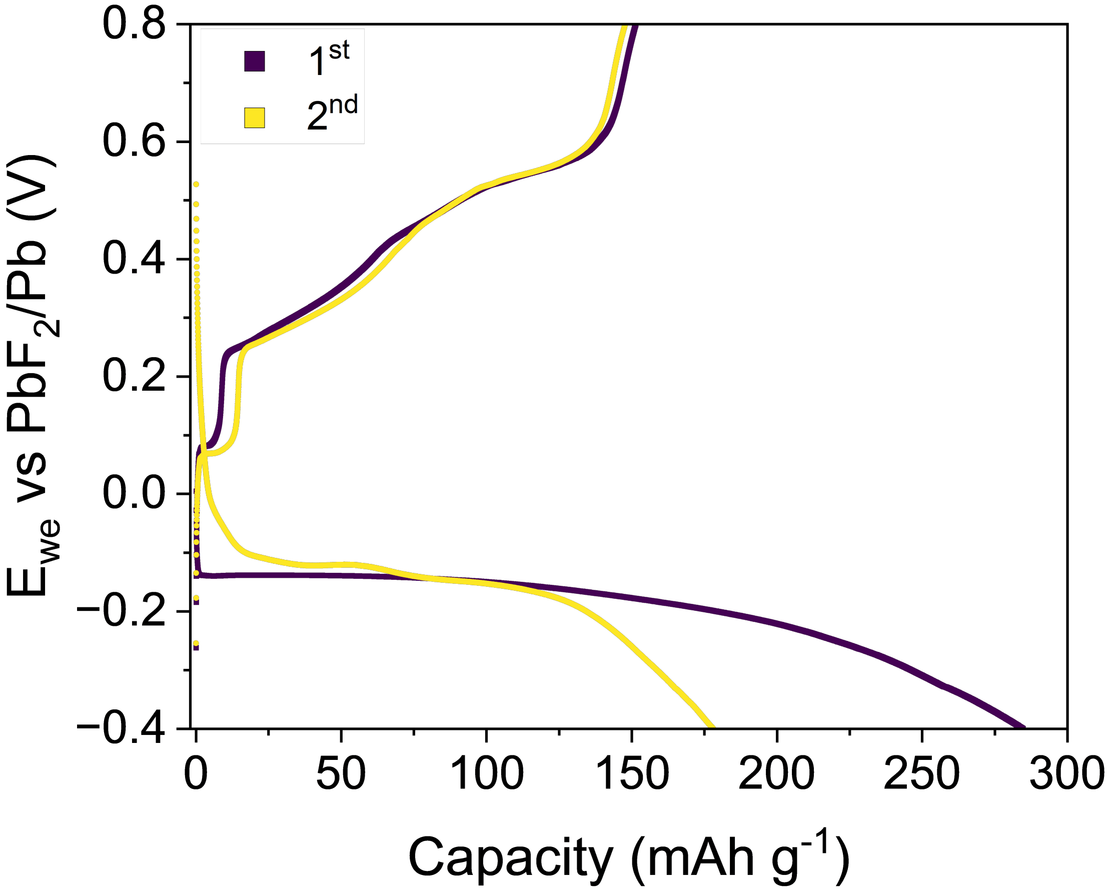

Figure S11: **Cycling reproducibility of BiF_3 vs Pb-PbF_2.**

The cycling behaviour of a second BiF_3 vs Pb-PbF_2 cell is in very good agreement with the cycling performance reported in Figure 5a.

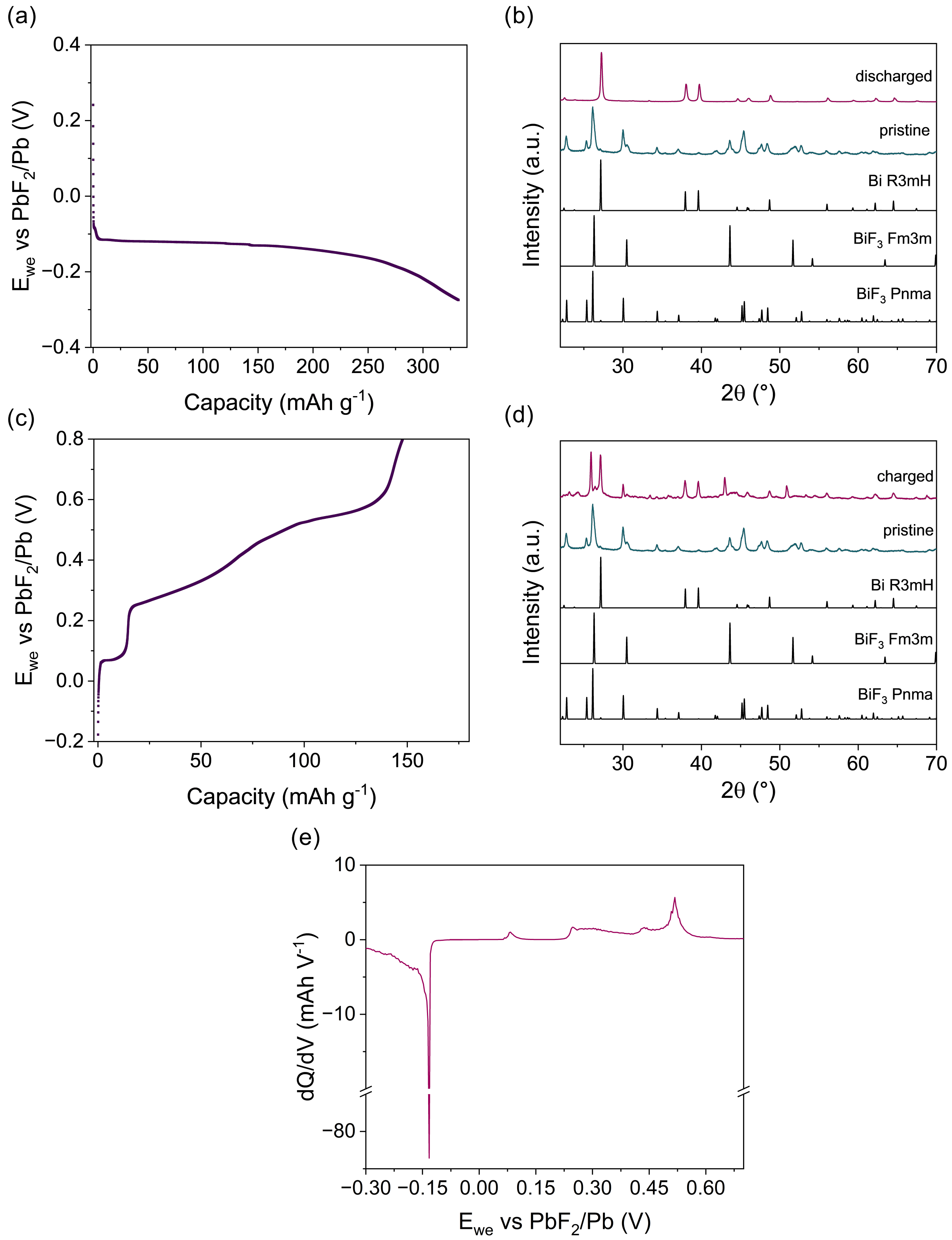

Figure S12: **Cycling behavior of BiF_3 electrodes** (a) Discharge profile of a BiF_3 electrode cycled versus Pb-PbF_2 with (b) its corresponding XRD. (c) Recharged BiF_3 electrode with (d) its XRD profile. (e) dQ/dV plot of the first cycle of the BiF_3|Pb-PbF_2 cell in Figure 5.

XRD results confirmed the occurrence of the conversion reaction during cycling. The pristine BiF_3 electrode comprises a mixture of cubic ($Fm\bar{3}m$) and orthorhombic (Pnma) crystal structures. At the end of the first discharge, the BiF_3 electrode converts to Bi metal, indicating that the observed capacity arises from the active material conversion (Figure S12a,b). Upon recharge, part of the Bi metal converts back to cubic BiF_3 proving

the reversibility of the conversion reaction. However, large peaks corresponding to Bi metal are still visible in the charged state, indicating incomplete conversion as evident from the lower than theoretical capacity obtained after recharging (Figure S12c,d). Furthermore, the several additional peaks present in the charged electrode indicate the formation of some unknown phases, possibly some intermediates, which require further investigation (Figure S12d). The cycling profile of BiF_3 appears asymmetric between charge and discharge (Figure S12e). The charge profile exhibits three plateaus, a short one at 0.07 V, a second one at 0.27 V and a third one at 0.52 V. The discharge profile displays a unique plateau at -0.12 V. A similarly asymmetric behaviour was observed by Yaokawa et al., who did not however explain the origin of it.[5] A mechanistic study is required to link each plateau to a specific crystallographic change. The coin cell setup presented in this work offers an ideal configuration for conducting such investigations through in-situ XRD.

Set-up	Counter electrode	Cycling rate	1st discharge capacity (mAh g-1)	1st charge capacity (mAh g-1)	Capacity retention 10th cycle (mAh g-1)	Reference
coin cell	Pb-PbF_2	C/20	284	180	173	this work
flooded cell	Pt	C/40	316	218	140	Kucuk et al.[6]
flooded cell	Pt	C/40	427	239	119	Konishi et al.[7]
coin cell	Mg	N/A	140	55	25	Mohammad et al.[8]
flooded cell	Pt	C/40	312	220	10	Konishi et al.[9]
flooded cell	Pt	C/40	228	180	N/A	Kucuk et al.[10]
flooded cell	Pt	C/40	340	117	N/A	Kucuk et al.[11]
flooded cell	Pt	C/40	215	100	N/A	Konishi et al.[12]
flooded cell	Pt	C/40	319	82	N/A	Konishi et al.[13]
2-electrode cell	Pb	C/360	110	60	N/A	Yaokawa et al.[14]

Table S1: The cycling performance of BiF_3 reported in this work is compared to what has been previously reported in the FIB literature. Cycling rate, discharge and charge capacity on the 1st cycle and capacity retention on the 10th cycle are selected as the parameters for the performance comparison. Additionally, the testing set-up and counter electrode used are reported for each study. The entries in red highlight the superior performance reported in this work compared to the other studies.

The BiF_3 cycling performance reported in this work does not only display the highest capacity retention at the 10th cycle but it is also recorded at a higher cycling rate compared to the previous studies reported in the literature. The majority of results reported in the literature do not achieve 10 cycles and those that do have a lower capacity retention. Additionally, it is worth noticing that the superior performance reported in this work is achieved in a full cell with a much lower electrolyte excess compared to the flooded cells used in the other studies.

		σ /Å	ϵ /kJ mol^{-1}	q /e
**MeOH**	**O**	3.12	0.71128	-0.683
	$\mathbf{C_M}$	3.5	0.276144	0.145
	**H**	-	-	0.418
	$\mathbf{H_M}$	2.50	0.12552	0.04
**TMA$^+$**	$\mathbf{N_1}$	3.25	0.71128	-
	$\mathbf{C_1}$	3.50	0.29288	0.07
	$\mathbf{H_1}$	2.50	0.12552	0.06
	$\mathbf{F^-}$	2.733	3.01248	-1.0
**Propionitrile**	$\mathbf{C_{1p}}$	3.30	0.27614	0.46
	$\mathbf{C_{2p}}$	3.30	0.27614	-0.02
	$\mathbf{C_{3p}}$	3.50	0.12552	-0.18
	$\mathbf{H_{1p}}$	2.50	0.12552	0.06
	$\mathbf{H_{2p}}$	2.50	0.12552	0.06
	$\mathbf{N_{1p}}$	3.25	0.12552	0.06

Table S2: Lennard-Jones parameters and charges from the OPLS-AA force field for methanol, tetramethylammonium cation (TMA^+), fluoride anion (F^-) and propionitrile.[17]

S0.6. Monte Carlo Simulations

Monte Carlo simulations were performed using the Empirical Potential Structure Refinement software (version 26.1) and visualised using the Aten packages.[15,16] The cubic simulation box of sides 43.12 Å contains 200 TMAF molecules and 624 methanol molecule to reproduce the experimental concentration of 10 molal (1:3.12 TMAF:MeOH molecular ratio) with atomic number density of 0.0916 atoms/Å^3. When propionitrile (PN) is added to make a 0.5 molal TMAF:MeOH:PN ternary mixture, the cubic simulation box of sides 76.11 Å contains 100 TMAF, 312 methanol molecules and 3450 propionitrile molecules to match the experimental density of 0.7885 g cm^{-3}, measured using a Anton Paar DMA 4100 density meter in an argon-filled glovebox at 30°C (<0.1 ppm O_2 and H_2O). The force field used are the OPLS-AA for methanol, fluoride anions, TMA cations and propionitrile with a potential cut-off of 18 Å.[17–19] Lennard-Jones potentials and charges are presented in Table S2. All molecules were built in Avogadro and the geometries were optimised using the MMFF94 force field for 1000 steps.[20,21] The simulations reached energy equilibration after 1,000 iterations, and a number of 10,000 configurations were accumulated in a trajectory file in order to extract the structural information of the two systems. In Figure S13 (a) the methanol H to fluoride anion partial distribution functions *g(r)* are plotted in the binary and ternary mixtures. The O-H$\cdots$ inter-molecular bonding distance is found at 1.58 Å and it does not change significantly when propionitrile is added. A close OH-F- interaction ($\sim$ 1.65 Å) has been seen previously for diluted aqueous solutions of ions via Monte Carlo simulations.[22] It is worth noting that this hydrogen bonding interaction is tight as it is found at short distances compared to other electrostatic intermolecular interactions in the liquid state.

The remarkable increase in peak intensity is attributable to micro-segregation of methanol in propionitrile. The microsegregation depends on the known tendency of methanol of forming linear and cyclic hydrogen bonding motifs, as it has been seen before in alcohol-water mixtures.[23,24] The microsegregation of methanol however confirms the strong solvation of fluoride ions by methanol, while propionitrile interacts with the anions by weaker van der Waals forces (Figure S13 (b) propionitrile N-F^-) such as dipole-ion and weak hydrogen bonding.

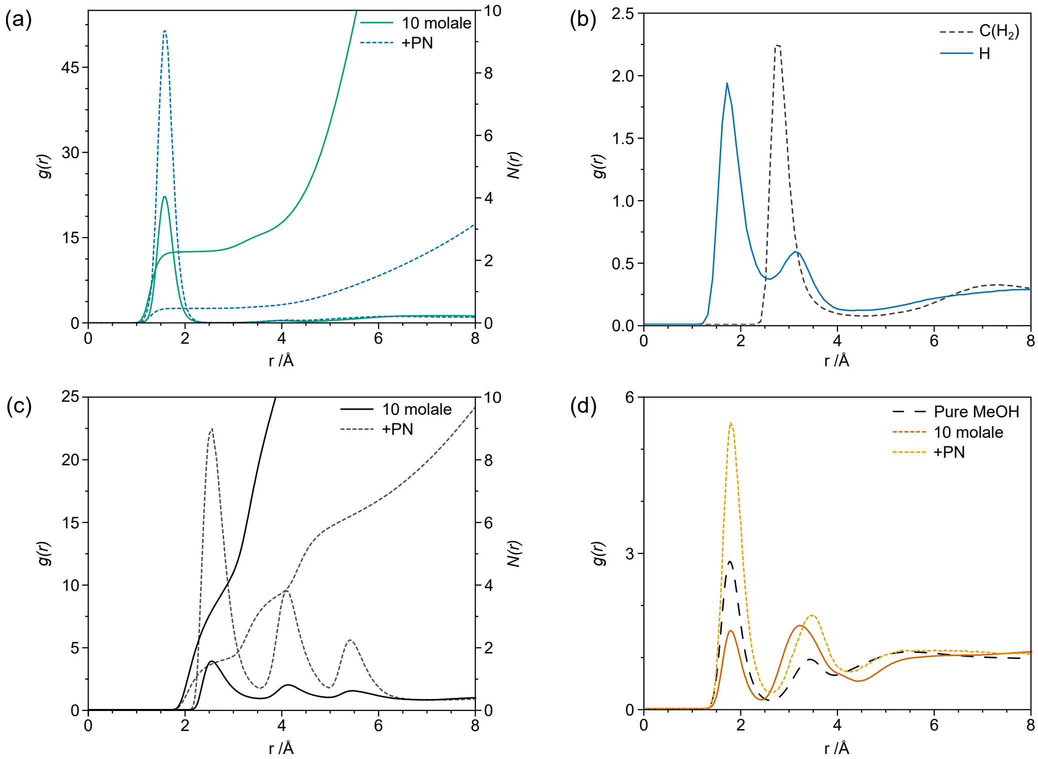

Figure S13: **Partial Radial Distribution Functions *g(r)* relative to (a) fluoride ion F^- and methanol proton H in presence (blue) and in absence of propionitrile (green). Note that the peak position does not change with the addition of the diluent; (b) fluoride ion F^- and propionitrile CH_2; (c) fluoride ion F^- and TMA^+ counterions in presence (dotted line) and absence (solid line) of propionitrile; (d) methanol proton H and methanol oxygen O in the pure liquid methanol, at TMAF 10 molal and in the ternary mixture. Note that the hydrogen bonding network of methanol is preserved.**

References

1. K. O. Christe, W. W. Wilson, R. D. Wilson, R. Bau, J.-A. Feng, Syntheses, properties, and structures of anhydrous tetramethylammonium fluoride and its 1:1 adduct with trans-3-amino-2-butenenitrile, J. Am. Chem. Soc 112 (1990) 7619–7625.

2. O. Alshangiti, G. Galatolo, G. J. Rees, H. Guo, J. A. Quirk, J. A. Dawson, M. Pasta, Solvent-in-salt electrolytes for fluoride ion batteries, ACS Energy Letters 12 (2023) 2668–2673. doi:10.1021/ACSENERGYLETT.3C00493.
URL https://pubs.acs.org/doi/full/10.1021/acsenergylett.3c00493

3. M. A. Nowroozi, O. Clemens, Insights on the behavior of conversion-based anode materials for fluoride ion batteries by testing against an intercalation-based reference cathode, ACS Applied Energy Materials 1 (2018) 6626–6637.

4. H. Konishi, T. Minato, T. Abe, Z. Ogumi, Reactivity of the anion acceptor in electrolyte: An important factor in achieving high electrochemical performance of a lead (ii) fluoride electrode in a fluoride shuttle battery, Journal of Electroanalytical Chemistry 871 (2020) 114103. doi:https://doi.org/10.1016/j.jelechem.2020.114103.

5. R. Yaokawa, T. Shiga, S. Moribe, K. Mukai, Evidence of a reversible redox reaction in a liquid-electrolyte-type fluoride-ion battery, RSC Advances 12 (2022) 31786–31791. doi:10.1039/d2ra05753k.

6. A. C. Kucuk, T. Yamanaka, Y. Yokoyama, T. Abe, Low-cost fluoride source for organic liquid electrolyte-based fluoride shuttle battery, Journal of The Electrochemical Society 168 (1) (2021) 010501. doi:10.1149/1945-7111/abd64d.

7. H. Konishi, A. C. Kucuk, T. Minato, T. Abe, Z. Ogumi, Improved electrochemical performances in a bismuth fluoride electrode prepared using a high energy ball mill with carbon for fluoride shuttle batteries, Journal of Electroanalytical Chemistry 839 (2019) 173–176. doi:https://doi.org/10.1016/j.jelechem.2019.03.028.

8. I. Mohammad, R. Witter, Testing mg as an anode against bif3 and snf2 cathodes for room temperature rechargeable fluoride ion batteries, Materials Letters 244 (2019) 159–162. doi:https://doi.org/10.1016/j.matlet.2019.02.052.

9. H. Konishi, T. Minato, T. Abe, Z. Ogumi, Electrochemical performance of a bismuth fluoride electrode in a reserve-type fluoride shuttle battery, Journal of the Electrochemical Society 164 (2017). doi:10.1149/2.0931714jes.

10. A. C. Kucuk, T. Abe, Borolan-2-yl involving anion acceptors for organic liquid electrolyte-based fluoride shuttle batteries, Journal of Fluorine Chemistry 240 (2020) 109672. doi:10.1016/j.jfluchem.2020.109672.

11. A. C. Kucuk, T. Minato, T. Yamanaka, T. Abe, Effects of libob on salt solubility and bif 3 electrode electrochemical properties in fluoride shuttle batteries, Journal of Materials Chemistry A 7 (2019) 8559–8567. doi:10.1039/c8ta10396h.

12. H. Konishi, T. Minato, T. Abe, Z. Ogumi, Improvement of cycling performance in bismuth fluoride electrodes by controlling electrolyte composition in fluoride shuttle batteries, Journal of Applied Electrochemistry 48 (2018) 1205–1211. doi:10.1007/s10800-018-1241-z.

13. H. Konishi, T. Minato, T. Abe, Z. Ogumi, Influence of electrolyte composition on the electrochemical reaction mechanism of bismuth fluoride electrode in fluoride shuttle battery, The Journal of Physical Chemistry C 123 (16) (2019) 10246–10252. doi:10.1021/acs.jpcc.9b00455.

14. R. Yaokawa, T. Shiga, S. Moribe, K. Mukai, Evidence of a reversible redox reaction in a liquid-electrolyte-type fluoride-ion battery, RSC Adv. 12 (2022) 31786–31791. doi:10.1039/D2RA05753K.

15. A. Soper, Empirical potential monte carlo simulation of fluid structure, Chem. Phys. 202 (2) (1996) 295–306.

16. T. G. A. Youngs, Aten—an application for the creation, editing, and visualization of coordinates for glasses, liquids, crystals, and molecules, J. Comput. Chem. 31 (3) (2010) 639–648.

17. W. L. Jorgensen, D. S. Maxwell, J. Tirado-Rives, Development and testing of the opls all-atom force field on conformational energetics and properties of organic liquids, J. Am. Chem. Soc. 118 (45) (1996) 11225–11236.

18. M. L. Price, D. Ostrovsky, W. L. Jorgensen, Gas-phase and liquid-state properties of esters, nitriles, and nitro compounds with the opls-aa force field, J. Comput. Chem. 22 (13) (2001) 1340–1352.

19. W. L. Jorgensen, J. P. Ulmschneider, J. Tirado-Rives, Free energies of hydration from a generalized born model and an all-atom force field, J. Phys. Chem. B 108 (41) (2004) 16264–16270.

20. T. A. Halgren, Merck molecular force field. i. basis, form, scope, parameterization, and performance of mmff94, J. Comput. Chem. 17 (5-6) (1996) 490–519.

21. M. D. Hanwell, D. E. Curtis, D. C. Lonie, T. Vandermeersch, E. Zurek, G. R. Hutchison, Avogadro: an advanced semantic chemical editor, visualization, and analysis platform, J. Cheminformatics 4 (1) (2012) 1–17.

22. J. Chandrasekhar, D. C. Spellmeyer, W. L. Jorgensen, Energy component analysis for dilute aqueous solutions of lithium (1+), sodium (1+), fluoride (1-), and chloride (1-) ions, J. am. Chem. Soc. 106 (4) (1984) 903–910.

23. S. Dixit, J. Crain, W. Poon, J. L. Finney, A. K. Soper, Molecular segregation observed in a concentrated alcohol–water solution, Nature 416 (6883) (2002) 829–832.

24. L. Dougan, S. P. Bates, R. Hargreaves, J. P. Fox, J. Crain, J. L. Finney, V. Réat, A. K. Soper, Methanol-water solutions: A bi-percolating liquid mixture, J. Chem. Phys. 121 (13) (2004) 6456–6462.
